# The association between implicit and explicit affective inhibitory control, rumination and depressive symptoms

**DOI:** 10.1038/s41598-021-90875-3

**Published:** 2021-06-01

**Authors:** Orly Shimony, Noam Einav, Omer Bonne, Joshua T. Jordan, Thomas M. Van Vleet, Mor Nahum

**Affiliations:** 1 School of Occupational Therapy, Faculty of Medicine, The Hebrew University, Mount Scopus, PO Box 24026, 9124001 Jerusalem, Israel; 2 Department of Psychiatry, Hebrew University-Hadassah Medical Center, Jerusalem, Israel; 3 Department of Psychology, Dominican University of California, San Rafael, CA USA; 4 Department of Research & Development, Posit Science Corporation, San Francisco, CA USA

**Keywords:** Attention, Cognitive control

## Abstract

Inhibitory control underlies one’s ability to maintain goal-directed behavior by inhibiting prepotent responses or ignoring irrelevant information. Recent models suggest that impaired inhibition of negative information may contribute to depressive symptoms, and that this association is mediated by rumination. However, the exact nature of this association, particularly in non-clinical samples, is unclear. The current study assessed the relationship between inhibitory control over emotional vs. non-emotional information, rumination and depressive symptoms. A non-clinical sample of 119 participants (mean age: 36.44 ± 11.74) with various levels of depressive symptoms completed three variations of a Go/No-Go task online; two of the task variations required either explicit or implicit processing of emotional expressions, and a third variation contained no emotional expressions (i.e., neutral condition). We found reductions in inhibitory control for participants reporting elevated symptoms of depression on all three task variations, relative to less depressed participants. However, for the task variation that required implicit emotion processing, depressive symptoms were associated with inhibitory deficits for sad and neutral, but not for happy expressions. An exploratory analysis showed that the relationship between inhibition and depressive symptoms occurs in part through trait rumination for all three tasks, regardless of emotional content. Collectively, these results indicate that elevated depressive symptoms are associated with both a general inhibitory control deficit, as well as affective interference from negative emotions, with implications for the assessment and treatment of mood disorders.

## Introduction

Major Depressive Disorder (MDD) is a costly, recurring chronic medical condition, ranked by the World Health Organization (WHO) as the single most burdensome disease worldwide with respect to total disability-adjusted years among midlife adults^1^. MDD is characterized by negative mood, anhedonia, sleep disturbances and lack of energy, which often lead to impairments in social and occupational functions^2,3^. Despite well-established and widely applied treatment modes, the majority of MDD patients relapse within two years of recovery, and over 80% of patients experience more than one depressive episode during their lifetime^4^.

Recent literature emphasizes deficits in inhibitory control, a component of executive function which refers to one’s ability to override a dominant or prepotent response^5^, as a key mechanism in depression^6,7^ and in dysphoria^8,9^. Inhibitory control can be described as comprised of three processes including interference resolution of task-irrelevant information, inhibition of no-longer relevant information in working memory and inhibition of prepotent responses^5,10^. Some theories suggest that deficits in inhibitory control lead to depressive symptoms via depressive rumination, a maladaptive form of emotion regulation^8,11,12^. By this view, it is not the initial activation of negative cognitions, but the inability to regulate the continued reactivation of these negative cognitions in order to alleviate negative mood, that increases the risk for depression. This, in turn, leads to enhanced processing of mood congruent (i.e., negative) information, resulting in persistent negative mood and the emergence or recurrence of depression^8,11,13^.

Collectively, these models suggest that depression is associated with a specific impairment in inhibiting *negative* information, and importantly, that depressive rumination mediates the link between the reduced inhibitory control over negative stimuli and depression^14–16^. Although there is generally strong evidence to support inhibitory deficits in depression^16–21^, it is not clear whether these deficits are limited to the processing of negatively-valenced information, nor whether non-clinical samples and at-risk populations exhibit similar vulnerabilities in inhibitory control^22^. Some authors further suggest that specific deficits in inhibiting emotional information may be present in non-clinical or at-risk cohorts, while more general inhibitory deficits (i.e., deficits that are evident irrespective of emotional valence) are seen in clinically depressed samples^22,23^. However, evidence regarding the nature of inhibitory deficits in non-clinical cohorts remains inconclusive^17,24^, as some studies have reported specific inhibitory control deficits over negative information only^25–27^, others report a more general or non-specific inhibitory control deficit^28^, and yet others find no inhibitory deficit at all in at-risk or non-clinical samples^24^. Given these mixed results, some authors have called for a more systematic investigation of the true nature of inhibitory control deficits associated with depressive symptoms^17,23,29^.

Given that inhibition is not a unitary concept, one potential source of the heterogeneity in results may be the type of inhibitory mechanism studied^11,30^. Of specific interest to the current study are deficits in the inhibition of prepotent responses in relation to depressive symptoms in non-clinical samples, which was proposed to be associated with rumination (see^31^). Prepotent inhibition has been reliably captured using a Go/No-Go (GNG) paradigm, in which the higher frequency of ‘Go’ stimuli creates a prepotent response pattern that must then be inhibited when infrequent ‘No-Go’ stimuli are randomly presented^32,33^. In addition, intra-individual variability of reaction times (RTs) to ‘Go’ stimuli has been associated with greater attentional fluctuations, or lack of attentional stability^34^. A variation of this task, the affective GNG task (AGN), requires responses to stimuli of a certain emotional valence (e.g., happy), while also requiring inhibition of responses to stimuli of an orthogonal emotional valence (e.g., sad)^35^. The AGN task has been shown to measure similar constructs as the original GNG task^32^, while also capturing the influence of emotional modulation of inhibitory processes^36^. Most studies that have used the GNG and AGN tasks in depressed individuals have collectively shown altered patterns of inhibitory control to specific emotional categories compared to healthy participants rather than a general inhibitory deficit irrespective of emotional valence^37–40^ (but see^41^). The few studies that have employed this paradigm in non-clinical or at-risk samples found reduced pre-potent inhibition to sad expressions in high ruminators^42^ and in adolescents who later developed depression^43^.

Depressive symptoms have been associated with mood-congruent attentional biases (e.g.,^6,44–46^), evident in enhanced implicit processing of, and attentional allocation to, negative emotions, while less attention is allocated to positive emotions^47^. Thus, targeting the emotional valence in the explicit decision process of the AGN may recruit different neural mechanisms compared with indirect (or implicit) emotional AGN task-related processing^48–50^. Task requirements for explicit emotion processing in the AGN task may more directly interfere with the inhibitory control processes^51^. Recently, Yu et al.^50^ have addressed this question using a modified version of the AGN task, which included explicit task blocks (i.e., response is required based on emotion category), and implicit task blocks, which required a response based on gender, regardless of emotion. Interestingly, their results show an inhibitory deficit over negative (sad) expressions in depression regardless of the task requirements, indicating that the deficit exists even without attentional allocation. Despite its significance, the study included a rather small clinical sample and did not include a neutral condition or condition with no emotional stimuli. Further investigation of implicit vs. explicit processing in relation to depressive symptoms is therefore needed.

Finally, the models cited above indicate the critical involvement of rumination in the association between inhibitory deficits and depressive symptoms: deficits in inhibitory control are hypothesized to cause difficulty in overcoming the prepotent tendency to ruminate, leading to recurrence or emergence of depression^8,11,52^. Indeed, several studies have provided compelling evidence for the association between rumination, inhibitory control and depression using various paradigms and populations (e.g.,^8,22,53,54^). Interestingly, however, the results are mixed in relation to the nature of the association between pre-potent inhibition and rumination in non-clinical or at-risk samples. For example, using the AGN task in a healthy, never depressed sample, Vanderhasselt et al.^42^ found that for high ruminators, more rumination correlated with inhibitory deficits over *negative*, but not positive expressions (see also^28^). However, another study failed to find an association between pre-potent inhibition (emotional or non-emotional) and rumination in a non-clinical sample^24^. A recent meta-analysis concluded that there is an association between prepotent inhibition and the pervasive nature of rumination regardless of the emotional content of the task (^31^; see also^55^). It is therefore still unclear whether the relationship between general and/or emotion-specific inhibition and depressive symptoms occurs through rumination, and whether this relationship is manifested differently given different attentional task demands.

In the current study, we investigated the association between depressive symptoms, inhibitory control and rumination by examining the involvement of emotional vs. non-emotional inhibitory control, while manipulating task demands to influence the allocation of attention. Specifically, we asked whether increased depressive symptoms are associated with deficits in inhibitory control irrespective of stimulus content or task requirements, and whether there is an indirect relationship of trait rumination between inhibitory control variations and depressive symptoms. To this end, we used three variations of the GNG task and manipulated the type of processing required (implicit vs. explicit) and the presentation of emotional stimuli (emotional vs. non-emotional). Thus, in the non-emotion (NE) task variation, participants responded based on gender, but the task included images of neutral expressions only, rather than emotional ones. In the explicit emotion (EE) task, participants inhibited response to neutral expressions and responded to emotional (sad and happy) expressions. Finally, in the implicit emotion (IE) task variation, participants responded based on the gender, rather than based on the emotional expression, which was a task-irrelevant dimension. This task variation allowed us to further assess attentional capture by the irrelevant emotional aspect of the stimuli.

Based on the literature cited above, we hypothesized that a generalized deficit in inhibitory control would be found in participants with more depressive symptoms and that this deficit may be enhanced when processing emotional information. We further examined whether this deficit would be more pronounced for the explicit, compared to implicit, emotional task variation. We further expected longer RTs and reduced RT variability for sad ‘Go’ trials in the IE task, which would indicate a mood-congruent attentional effect. Finally, as an exploratory step, we tested whether there was an indirect relationship of rumination between inhibitory control and depression and hypothesized that this relationship will likely be strongest for the IE task variation, which uses similar mechanisms of biased attentional capture.

## Results

### Characterization of study sample

A sample of 119 adults was recruited for the study via two online platforms: Amazon Mechanical Turk (MTurk; n = 45) and Prolific Academic (n = 74). Five of the 119 participants were excluded from further analyses due to the following constraints: responses from 2 participants were not recorded for the inhibitory control tasks, 2 participants performed the tasks on tablets instead of using a PC/laptop as requested, and one participant experienced technical problems operating the experiment.

A total of 114 adult participants (age range: 18–65 years; Average: 36.44 ± 11.74 years) completed the study, 70 of them (61.4%) were female. The sample was not a clinical sample. However, according to PHQ-9 scale classification, 32 participants (28.9%) were classified as having minimal depression, 24 (21.1%) as having mild depression, 23 (20.2%) as having moderate depression, and 34 (29.8%) as having moderately severe to severe depression (PHQ-9 scores of 15 and over). A significant correlation was found between depression (PHQ-9) and rumination (RRS; r = 0.784, *p* < 0.001). Demographic information is presented in **Table ****1**. Additional information regarding mental health status and medication history of the group can be found in Supplemental Table 1.

**Table 1:** Demographic information of study sample.

Depression level (PHQ-9 category)	PHQ-9 score range	N	Gender (% F)	Age (y)
Minimal	0–4	33	63.6	40.9 ± 11.6
Mild	5–9	24	54.2	37.3 ± 9.8
Moderate	10–14	23	56.5	33.1 ± 11.97
Mod. severe	15–19	21	71.4	33.6 ± 10.2
Severe	20–27	13	61.5	34.1 ± 14.9
**Total**		**114**	**61.4**	**36.4 ± 11.7**

### Emotional vs. non-emotional inhibitory control (GNG) tasks

**Figure 1:**
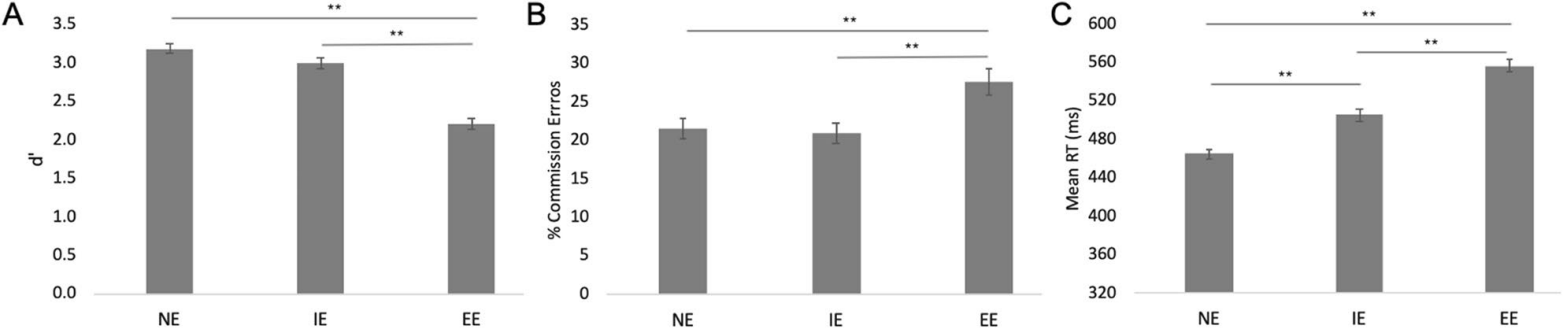
Comparison between the three GNG task variations: no-emotion (NE), implicit emotion (IE) and explicit emotion (EE). (**A**) d’; (**B**) Percentage of commission errors for No-Go trials; (**C**) Mean reaction time (RT) in ms; Averages and standard errors of mean (SEMs) are presented.

Participants completed the 3 GNG task variations during the study in random order. In order to examine differences in task difficulty between the three task variations, we calculated three different measures: d prime (d’), commission error rate (errors for No-Go stimuli), and response times (RTs) for Go stimuli. Analysis of d’ revealed a statistically significant difference between the 3 tasks (NE, IE, and EE; F(2,112) = 64.0, p < 0.001; partial η^2^: 0.27). Specifically, difficulty of the EE task was higher (d' = 2.2 ± 0.7) compared to the two other tasks, while there was no difference in difficulty between the NE and the IE tasks (d ' = 3.2 ± 0.6 and 3.0 ± 0.7 for NE and IE, respectively; see **Fig. ****1****A**).

In addition, there was a statistically significant difference in commission errors between the three tasks (F(2,113) = 15.48, p < 0.001; partial η^2^: 0.12), with significantly more commission errors for the EE task (27.7 ± 18.3%), but no difference in commission errors between the NE and IE tasks (21.5 ± 1.3% and 21 ± 1.3%, respectively; **Fig. ****1****B**). RT analysis for ‘Go’ trials similarly showed significant differences between the tasks (F(2,112) = 161.9, p < 0.001; partial η^2^: 0.59): average RT was shortest for the NE task (464.8 ± 5.5 ms) and longest for the EE task (556.3 ± 6.5; **Fig. ****1****C**). Collectively, these results show that the EE task was more difficult than the two other task variations. Finally, there was a statistically significant difference in bias to respond (the criterion, ‘c’) between the three tasks (F(2,112) = 19.51, p < 0.001), with larger bias found for the NE task (− 0.70 ± 0.30) and smaller for the IE (− 0.59 ± 0.33) and the EE (− 0.41 ± 0.41) tasks. The larger bias for the easier NE task indicates that participants were more liberal in their tendency to respond. In contrast, the more difficult EE task led participants to make more conservative choices, resulting in a smaller response bias.

### Association between depressive symptoms and inhibitory control abilities

**Figure 2:**
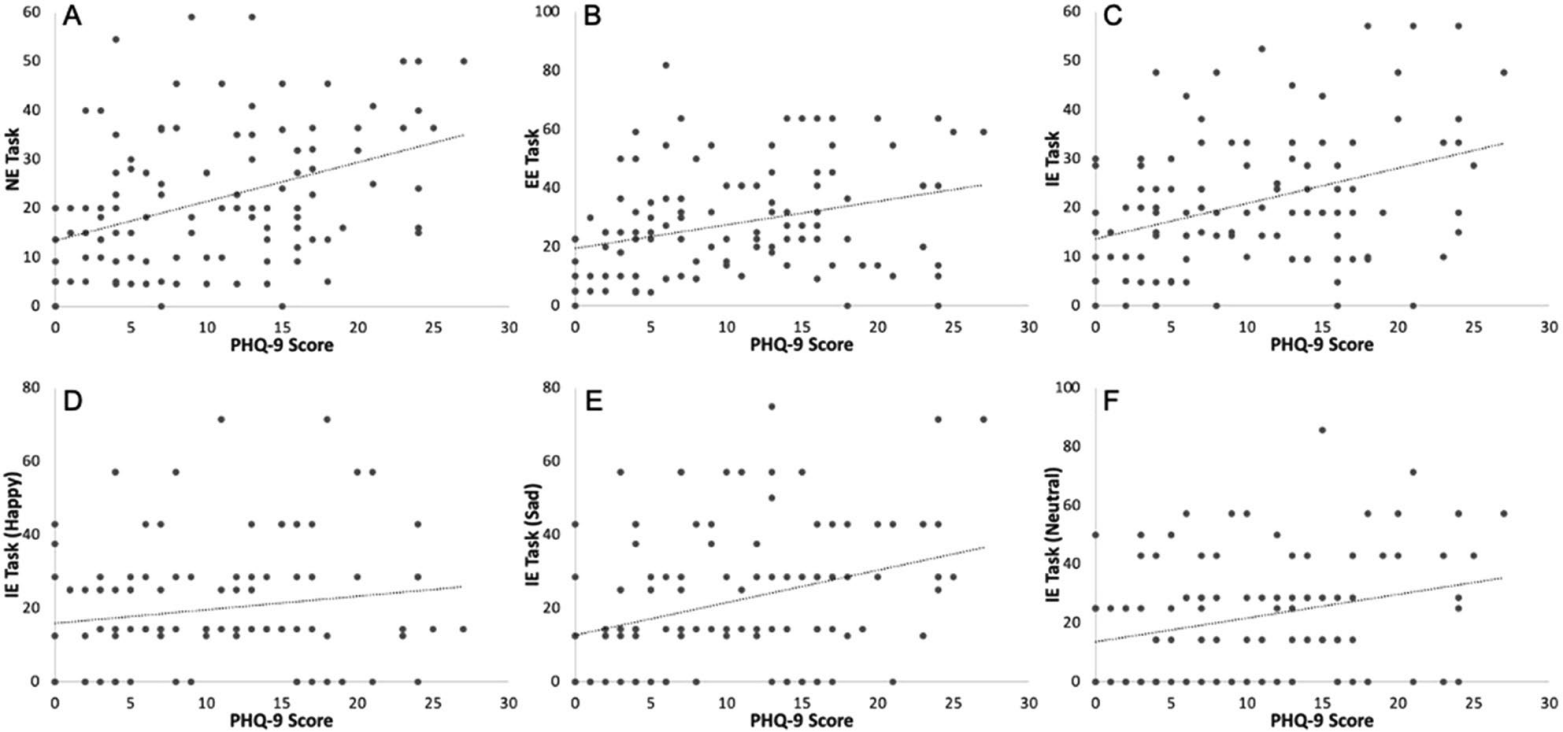
% Commission errors as a function of level of depression. (**A**–**C**). Pearson Correlations between PHQ-9 scores and % commission errors for the NE (**A**), EE (**B**) and IE (**C**) tasks. (**D**,**E**). Pearson correlations between PHQ-9 and %commission errors for the IE task, split by emotion: happy(**D**), sad (**E**) and neutral (**F**).

We next asked whether inhibitory control ability (i.e., % commission errors) differs as a function of depression level for all GNG tasks. These results are summarized in Fig. 2. There was a significant correlation between level of depressive symptoms and inhibitory control on all task variations (NE task: r = 0.4, *p* < 0.001; EE task: r = 0.311, *p* < 0.001; IE task: r = 0.377, *p* < 0.001).

For the IE task, we further examined whether this correlation was present for all three emotion types. We found a significant correlation between depressive symptoms and commission errors for sad (r = 0.334, *p* < 0.001) and neutral expressions (r = 0.285, *p* < 0.003), but not for happy expressions (r = 0.157, *p* = 0.095).

Finally, we examined the association between level of depressive symptoms and RT and RT variability (SD RT) to Go trials in all 3 tasks, as an indicator of attentional fluctuations and instability. There was a weak inverse association between the PHQ-9 score and mean RT for the NE task variation (r = − 0.185, *p* = 0.049), but not for any of the other two tasks (IE: r = − 0.086, *p* = 0.365; EE: r = 0.091, *p* = 0.337). However, PHQ-9 score was inversely associated with the RT variability of sad stimuli in the IE task (r = − 0.233, p = 0.013), such that those with higher levels of depressive symptoms had lower variability in RT for sad stimuli. No associations were found between PHQ-9 and RT variability of both happy (r = − 0.006, p = 0.95) and neutral (r = − 0.017, p = 0.86) expressions of the IE task.

### Predictability of depressive symptoms by inhibitory control abilities

We next performed a linear regression in order to examine the proportional contribution of demographic variables (i.e., age) and inhibitory control in explaining the variance of depression (PHQ-9 score). We first verified that the correlations between the predictors (commission errors of NE, EE and IE) are not higher than 0.7, which was indeed the case (NE-IE: r = 0.61, *p* < 0.001; NE-EE task r = 0.57, *p* < 0.001; IE-EE task r = 0.59, *p* < 0.001), confirming there is no multicollinearity. We then performed a linear regression, using age and all 3 inhibitory control parameters as predictors. The results are given in **Table ****2**. The model accounted for 21.5% of the variance of the PHQ-9 score (F = 7.45, *p* < 0.001), and only inhibitory control in the non-emotional (NE) task ($\beta$ = 0.24; t = 2.11, *p* = 0.037) independently contributed to the PHQ-9 variance, while the contribution of IE and EE inhibition was non-significant.

**Table 2:** A linear regression analysis for prediction of depression level (PHQ-9 total score).

Variable	$\beta$	F/t	p	R^2^
Overall model		7.45	< .001	.215***
Age	− .16	− 1.86	.066	
NE inhibition	.24	2.11	.037*	
IE inhibition	.18	1.56	.122	
EE inhibition	.02	.174	.86	

*p < 0.05, ***p < 0.001.

Finally, we performed a linear regression to examine whether inhibitory control of specific emotions within the IE task (neutral, sad, and happy) explains the variance in depression scores (PHQ-9). In this model, which accounted for 18.8% of the variance in depression scores, inhibition to implicit sad and neutral expressions, but not happy expressions, were significant contributors (see **Table ****3**).

**Table 3:** A hierarchical linear regression analysis for prediction of depression level (PHQ-9 total score) from IE task parameters.

Variable	$\beta$	F / t	p	R^2^
		6.6	.000	.188***
Age	− .11	-2.08	.039*	
IE inhibition sad	.09	2.45	.016*	
IE inhibition neutral	.077	2.37	.020*	
IE inhibition happy	− 0.009	− .21	.831	

*p < 0.05, ***p < 0.001.

### Indirect effect of rumination between inhibitory control and depressive symptoms

**Figure 3:**
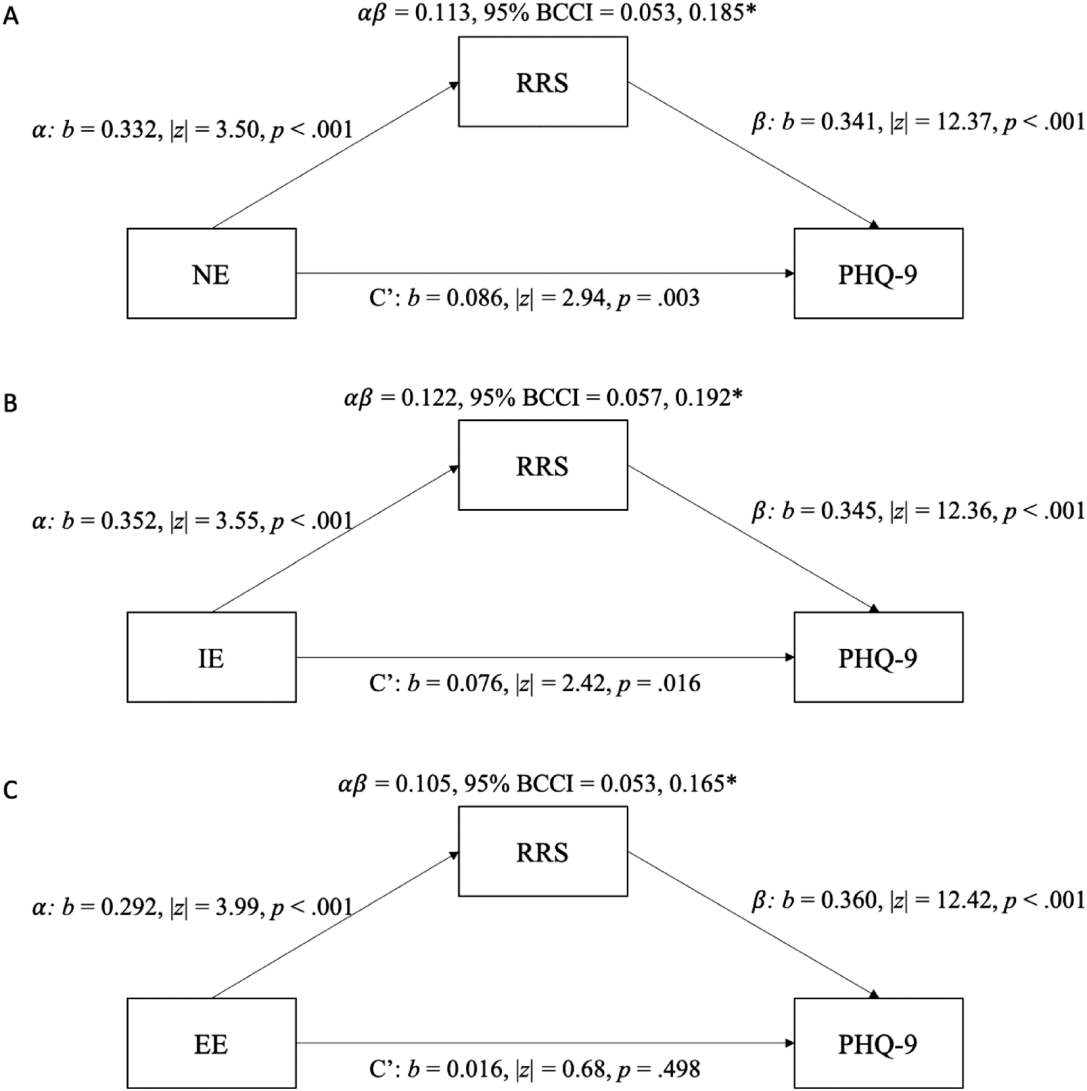
Analysis of indirect effects for RRS between NE inhibition (**A**), IE inhibition (**B**) and EE inhibition (**C**) and depression (PHQ-9 total score).

As an exploratory step, we tested whether there was an indirect relationship of RRS between IC and depression severity. Because our data was cross-sectional, we could not formally test for mediation, as these models can generate biased estimates^56^. Nevertheless, because of the strong theoretical basis for involvement of rumination^8,11^, we ran these analyses with these caveats in mind. Cross-sectional indirect effects analyses were conducted with the product of coefficients approach, with 5,000 bootstrapped replications of the indirect effect^57^. We ran three separate models, one for each IC paradigm (NE, IE and EE). Results are displayed in **Fig. ****3**. There was a significant indirect effect with NE ($\alpha \beta$ = 0.113, 95% Bias-Corrected Bootstrapped Confidence Interval [BCCI] = 0.053, 0.185; **Fig. ****3****A**), IE ($\alpha \beta$ = 0.113, 95% Bias-Corrected Bootstrapped Confidence Interval [BCCI] = 0.057, 0.192; **Fig. ****3****B**), and EE ($\alpha \beta$ = 0.105, 95% Bias-Corrected Bootstrapped Confidence Interval [BCCI] = 0.053, 0.165; **Fig. ****3****C**). The proportion of the relationship between IC and depressive symptoms through rumination was 56.75% for NE, 61.69% for IE and 86.51% for EE.

Even when accounting for the indirect effect of RRS, there was still a direct effect for NE (b = 0.086, |z|= 2.94, *p* = 0.003) and IE (b = 0.076, |z|= 2.42, *p* = 0.016), but not EE (b = 0.016, |z|= 0.68, *p* = 0.498), suggesting that the relationship between EE and severity of depressive symptoms occurs primarily through RRS. The results remained the same even when including age as a covariate in the models.

Finally, as a sensitivity analysis, we examined whether the indirect effect of RRS was driven by a specific subscale (i.e., brooding, reflection and depression) by running additional indirect effects models where the specific subscales served as the indirect effect. All subscales were statistically significant as indirect effects, and there was no evidence that any specific subscale had a larger effect than others, as all bootstrapped 95% confidence intervals overlapped with one another (range: ab = 0.039, 95% BCI = 0.003, 0.094; ab = 0.137, 95% BCI = 0.067, 0.214; see Supplementary Table 2 for full results).

## Discussion

In the current study, we examined the relationship between inhibitory control, rumination and the severity of depressive symptoms using three variations of the GNG task, collectively manipulating task requirements for explicit, implicit or no emotional processing. We found a significant and strong correlation between the magnitude of depressive symptoms and inhibitory control (commission errors for No-Go stimuli), regardless of task variation, such that more depressed participants had worse inhibitory control. However, examining the individual emotions within the implicit emotion (IE) task, we found a correlation between levels of depressive symptoms and inhibition for implicit sad and neutral, but not for happy expressions. Furthermore, higher levels of depressive symptoms were associated with lower trial-by-trial variability in RT for sad ‘Go’ expressions in the IE task. A regression model incorporating inhibition of sad and neutral expressions within the IE task explained 18.8% of the variance in depression scores. Finally, an indirect effect of trait rumination was found between inhibitory control and depressive symptoms for all three task variations, but only for EE this relationship occurred primarily through rumination.

The strong correlation between self-reported depressive symptoms and inhibitory control performance for all three task variations lends support for a more *general*, rather than emotion-specific, inhibitory control deficit in this non-clinical sample. Even when no emotional information was included, as in the NE task variation, participants with more depressive symptoms had reduced inhibitory control ability. These results are consistent with a handful of other studies showing a general and non-emotion-specific inhibitory or cognitive control deficit in patients with depression or dysphoria^20,41,58^ and in non-clinical samples at risk for depression^14,55^. For example, Quinn et al.^41^ used a general (non-emotional) GNG task and found inhibitory control deficits in those with melancholic depression compared to those with non-melancholic depression or healthy controls.

However, several theoretical accounts actually suggest a specific deficit in inhibiting *negative*, rather than general or positive information in depression or dysphoria or in non-clinical samples at risk for depression^6,59^. This deficit has been suggested to give rise to maladaptive emotion regulation strategies, such as depressive rumination, which has been consistently implicated as a key mechanism in depression^60^. Indeed, many studies report a deficit in various forms of inhibition over negative, but not of positive information^25,61,62^ (see reviews in^16,18^). Specifically, studies using the *explicit* version of the affective GNG task, which requires response based on the emotional content of the stimulus (e.g., sad vs. happy) often report specific deficits in prepotent inhibition to sad compared with happy stimuli in depression^38,50,63^ and in non-clinical samples^25,42,43^. Given that most studies of prepotent inhibition deficits in non-clinical populations did not include a direct comparison of affective vs. non-affective (i.e., emotionally-neutral) conditions, more studies are needed in order to understand the exact nature of these deficits in non-clinical samples (but see^20,24,25^).

Nonetheless, although our results may point to a more *general* inhibitory control deficit, a closer examination of the implicit emotion GNG task when emotional information is irrelevant to task performance revealed a pattern which is consistent with a negative-valence deficit in individuals with more depressive symptoms. Specifically, when participants were required to attend to gender and thus, could ignore the irrelevant facial expression, more depressed individuals exhibited inhibitory deficits for sad and neutral, but not for happy expressions. In addition, inhibition of sad and neutral expressions was a significant predictor of the level of depression (PHQ-9 scores). These results are generally consistent with the ‘affective interference hypothesis’ proposed by Siegle and colleagues^46^ and with an affective processing bias^64^. According to this hypothesis, irrelevant negative content attracts the attention of dysphoric individuals, at the expense of attending to the task-relevant aspects of the stimulus; resulting in impaired performance when the negative emotional valence is task irrelevant, but not when it is task relevant. Thus, participants with more depressive symptoms may have difficulty disengaging attention away from irrelevant negative information^6,60^. Using a lexical decision task, Siegle et al.^46^ found that dysphoric participants had longer RTs in cases of irrelevant negative information, and shorter RTs in cases of relevant negative information. Studies using other paradigms, which require inhibition of task-irrelevant inhibitory materials, such as the Negative Affective Priming (NAP) task^65^, the Stroop inhibition task^25,66^ or the emotional flanker task^62^ similarly reported specific deficits in inhibition of negative expressions, indicating an inability to ignore or suppress negative information (see reviews in^16,18,67^).

Interestingly, however, the inhibitory deficit characterizing individuals with more depressive symptoms in the IE task variation in the current study was found not only for implicit sad expressions, but also for neutral expressions. This may be evidence of a negativity bias or tendency to interpret neutral emotions as more negative, which is often associated with depression. Indeed, a similar effect was found in a previous study by our group, in which participants with higher levels of depressive symptoms exhibited more confusion between sad and neutral expressions in an emotion matching task^44^ (see also^68^). Conversely, based on the affective interference hypothesis, we would also expect to find a *facilitation* effect (i.e., faster RTs) for negative stimuli in the explicit emotion (EE) task, which was not shown in our study.

Our results show no significant correlations between RT in ‘Go’ trials and level of depressive symptoms for all three task variations (excluding a weak inverse association with RT for the NE task). Results in the literature are mixed, in that some studies examining performance in emotional inhibitory control tasks report faster RTs for positive stimuli in participants with depression^32,35,69^, while others report faster RTs for negative stimuli in more depressed individuals (e.g.^38,63,65^) (the latter is consistent with a facilitated response to negative or mood-congruent stimuli). The fact that no RT-specific effects were found in our study could result from the fact that the study sample was a non-clinical sample, whereas other studies that demonstrated this effect often used clinical samples. However, we did find a significant correlation between RT variability for sad expressions in the IE task and level of depression: individuals with higher levels of depressive symptoms had lower RT variability for ‘Go’ stimuli with sad expressions, but not for neutral or happy expressions. No such correlation was found for the EE task variation, in which the emotional category was task relevant. RT variability in GNG tasks is often considered a measure of sustained attention and attentional stability^70^. Using this metric, we can conclude that individuals with more depressive symptoms show higher levels of attentional stability to implicit, rather than explicit, negatively-valenced stimuli, for which a negative-bias already exists (see also^38^). Thus, processing of sad expressions is amplified in depression both by difficulty inhibiting sad ‘No-Go’ stimuli, and by increased sustained attention to sad ‘Go’ stimuli, which suggests a pre-primed and almost automatic mood-congruent response.

In addition, using an exploratory analysis, we found an indirect effect of trait rumination between inhibitory control and depression for all three GNG task variations, regardless of task demands or emotional content. Models by Joormann and others have emphasized the mediating role of rumination, suggesting that inhibitory deficits give rise to rumination and negative affect, which in turn leads to depressive symptoms^12,19,52^. However, results in the literature are mixed regarding the potential connection of rumination to either general or negative-specific inhibition. For example, Vanderhasselt et al.^42^ found that high brooders have difficulty specifically inhibiting sad stimuli (see also^54^). In contrast, other studies have found an indirect or mediating effect for rumination between *general* attentional and inhibitory control deficits in depression and in at-risk samples (e.g.,^20,53,55^; see also recent meta-analysis by Yang et al.^31^). Given the fact that we used a cross-sectional study design, our results regarding the indirect effects of trait rumination should be interpreted with caution. Still, they are in line with those studies that have linked general inhibitory deficits to rumination and lend further support to models which suggest that lack of inhibition may lead to rumination over negative content in depression, which in turn leads to sustained negative mood and depression. Interestingly, however, our results further suggest that direct associations between inhibition and depressive symptoms also exist for the NE and IE task variations, but not for the EE task variation. For the EE task, which requires explicit emotional processing, the association exists only via rumination. Future studies should further examine this association in clinical samples using longitudinal designs to test true models of mediation.

The results of our study have some implications for individuals with dysphoria or who are at risk for depression, as they help understand the underlying deficits in inhibitory control mechanism associated with depressive symptoms. Specifically, a deficit in inhibitory control of implicit negative emotions may be an objective tool for assessing symptoms related to depression, which may also have validity beyond or in addition to the standard self-report measures often used to assess depression. Some studies indicate that these cognitive deficits are even apparent *before* other depressive symptoms emerge and are thus useful for the prediction of depression^44,54^. Although the design of the present study does not permit true prediction, we observed that inhibitory control was a predictor of depressive symptoms in a regression model. In addition, targeted cognitive training which specifically addresses the inhibitory control deficits found here may be useful for the treatment and/or prevention of depression. This, of course, requires further investigation of causality. In recent years, various protocols of cognitive control training have been tested for depression, with some promising results^71–73^. Interestingly, some authors found that emotion-specific cognitive control training is more effective than general training, which has no emotional content (e.g.^74^). Based on the results of the current study, training which involves emotions as an implicit dimension within an inhibitory control paradigm may be beneficial for depression or dysphoria, in combination with other treatment approaches. The extensive use of mobile technology^75^ and the simplicity of the GNG paradigm make its implementation as an ecologically valid, mobile training approach feasible.

Our study has several limitations that should be taken into account when interpreting the results. A main limitation of the study is related to the fact that data collection was completed remotely, using participant’s personal devices/platforms. Despite the many benefits of these platforms^76^, and the various data-assurance protocols we applied in order to make sure that the data collection was valid^77,78^, participants still completed the tasks remotely and not under direct supervision in our lab. External factors, such as noise, distractions, Internet connectivity, etc. may have impacted task performance^79^. Further, we currently lack a direct comparison between home-based and lab-based versions of the tasks used in the study. In addition, information regarding co-morbidities or other conditions, such as the presence of attention deficit disorder (ADD/ADHD), as well as additional demographic variables, which may predict deficits in executive functions^80^, was not collected. Moreover, the study sample is probably not representative of the general population, given the unique profile of MTurk and Prolific users^81^, as well as the relatively higher rates of depression and anxiety in this population^78^. Additionally, as there were differences in the difficulty between the EE GNG task and the two other tasks, both in terms of commission errors and in response times, one cannot rule out the influence of task difficulty on the results. Future studies should address these issue in order to validate results when difficulty level is equated across tasks. Finally, although we observed a significant indirect effect of rumination between IC and depression severity, these models were significantly limited as they were cross-sectional. Cross-sectional tests of indirect effects can be misleading and provide biased estimates of indirect effects even under ideal conditions^56,82,83^. This finding therefore should be interpreted with caution, and future longitudinal studies are needed to formally examine whether rumination mediates the relationship between IC and depression severity.

In conclusion, our study points to the complex mechanisms of inhibitory control, which operate in relation to depressive symptoms. Although there seems to be a *general* inhibitory control deficit in individuals with more depressive symptoms, implicit processing of task-irrelevant emotions showed that this deficit is amplified for sad and neutral expressions, and more sustained attention (lower RT variability) towards implicit negative information. Future well-designed studies could help to further illuminate the significance of these inhibitory control mechanisms in depression, as well as determine the utility of examining inhibitory control functions to predict the future onset of depression in healthy adults.

## Methods

### Participants

A sample of 119 adults were recruited for the study via two online platforms: Amazon Mechanical Turk (MTurk; n = 45) and Prolific Academic (n = 74). The study was conducted online and without knowing the participants identity. Inclusion/exclusion criteria for study participants via self-reports were: (a) Be registered as workers in one of the platforms; (b) Age range: 18–65; (c) Native speakers of English; (d) No history of traumatic brain injury based on self-reports; and (e) No history or current personality disorder and/or schizophrenia based on self-reports.

To ensure cleanliness of the data, and following suggestions in recent publications related to studies using online platforms^77,84^, the following selection criteria were employed: (a) Residents of the United States (for MTurk participants) or United Kingdom (for Prolific Academic participants) ; (c) Have the "Master's Certification"—workers that consistently demonstrated a high degree of success in performing a wide range of Human Intelligence Tasks (HIT) across a large number of requesters; (d) Approval Rate of >  = 95% of their HITs. This is a System Qualification index, which helps us select participants who have consistently produced high quality tasks. The “Hit Approval Rate” represents the proportion of completed tasks that are approved by Requesters in different tasks. (e) Over 1000 HITs approved. This means that only participants that completed at least 1000 approved tasks in the platform will have access to the current experiment, allowing us to further select qualified participants who produce reliable data. In addition, in order to ensure that the participants are humans and not computer bots, we included a few verification questions throughout the experiment^85^.

### Experimental procedures

Approval was obtained from the Ethics Committee for Non-Medical Research in the Faculty of Natural Sciences, Medicine and Dentistry, The Hebrew University of Jerusalem. The study was conducted in accordance with the Declaration of Helsinki. All experiments were run on two online crowdsourcing platforms: Amazon’s MTurk and Prolific Academic (for information about experiments development, see Supplementary Material 1).

Informed consent was obtained from all study participants before beginning any study related procedures. Participants who signed up for the experiment were first required to provide an informed consent and complete a demographic and medical questionnaire, followed by self-report questionnaires to assess mental health status. Participants then completed 3 variations of an inhibitory control task (non-emotional, explicit emotion, implicit emotion; see below). The entire experiment took a total of 30 min. Participants were asked to use their desktop/laptop to complete the study, and not their mobile phones or tablets, since the tasks best fitted the computer screen size.

Collected data was transferred to a secure data store server where unidentifiable information is stored. Access to data was given only to the research staff members who were able to check the response rate in real time and detect data absorption problems. At the end of the experiment, participants received monetary compensation of 6$.

### Research tools

#### Mental health self-report measures

We used the following self-report measures to assess depression and rumination:

##### Patient health questionnaire, 9-item (PHQ-9^86^).

A standardized 9-item self-report questionnaire assessing DSM-V-TR symptoms of depression experienced in adults in the two weeks preceding the administration. The participant should indicate how often they experience the depressive symptoms that described in each item with a scale of 0 (not at all) to 3 (nearly every day). The total score of the questionnaire (range 0–27) indicates the severity of depression, with a higher score indicates higher symptom severity. Participants take 3–5 min to complete the questionnaire. The PHQ-9 is the most commonly administered self-report tool for depression. It has good diagnostic and psychometric properties, with high positive predictive value for depression (50% for PHQ-9 scores of 15 and over, and 75% for scores of 20 and over)^86,87^. In the current study, the scale had excellent internal consistency (Cronbach’s $\alpha$=0.924).

##### Ruminative responses scale (RRS^88,89^)

A standardized, validated 22-item self-report questionnaire used to assess level of rumination experienced in the 2 weeks preceding administration. The scale has 3 factors: depression (D), brooding (B) and reflection (R)^88^. We derived both a total score summing all items as well as the 3 individual factors. Excellent internal consistency was found for this scale in our sample (Cronbach’s $\alpha$=0.959).

#### Computerized inhibitory control tasks (GNG)

We used 3 variations of the GNG task, which is a widely used continuous performance test (CPT) to assess inhibitory control by measuring prepotent response inhibition^32,33^. In the current experiment, participants completed the three task variations (non-emotional, explicit emotion and implicit emotion) in random order, as described below.

**Figure 4:**
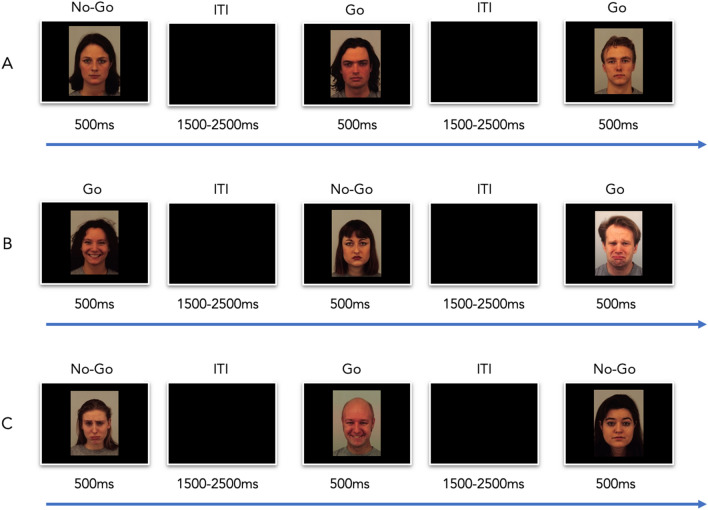
Examples of trials of the 3 GNG task variations. (**a**) NE variation: participants should respond to male faces and withhold response to female faces. All faces are with neutral expressions; (**b**) EE variation: participants should respond to emotional faces (happy or sad expressions) and withhold response to neutral facial expressions; (**c**) IE task variation: participants should respond to male faces and withhold response to female faces (regardless of the facial expression, which can be happy, sad or neutral).

On each task variation, participants were serially presented with pictures of faces and were asked to respond as quickly and as accurately as possible to frequent stimuli (80% of stimuli; non-targets, foils) and withhold response to rare target stimuli (20% of stimuli; see **Fig. ****4**). Each block of the task included a total of 100 trials. On each trial, a stimulus was presented on the screen for 500 ms followed by an inter-trial-interval (ITI) which was randomly jittered between 1500–2500 ms. The response window was of 1000 ms—the 500 ms of the stimulus presentation and the first 500 ms of the ITI. The sequence of stimuli within a block was pseudo-randomized for each run, and we made sure that there were never two consecutive targets. No feedback was given for participants on their responses.

Task instructions were given at the beginning of each block, followed by 10 practice trials, to ensure that participants understand the task. Each block took 3 min to complete. A standard inhibitory control metric was derived from each block, calculated as percent of commission errors (incorrectly responding on No-Go trials; see^63,90^ for similar outcome measures).

#### Stimulus set

The facial expression stimuli included in all 3 task variations were chosen from the Karolinska Directed Emotional Faces (KDEF^91^). The selected set consisted of 35 adult male and 35 adult female faces, all showing equal numbers of the three different expressions (happy, sad, and neutral), resulting in 210 stimuli in total. Face stimuli were presented on a black background, with each picture was at a size of 239/324 pixels.

The following task variations were used:

Non-emotional (NE): participants were asked to respond to pictures of male faces (‘Go') and withhold response to female faces (‘No-Go'). For this task variation, we only used pictures showing neutral facial expressions: 80 male faces and 20 female faces.

Explicit emotion (EE): participants were asked to respond to *emotional* expressions (sad or happy expressions, 80% of stimuli; ‘Go') and withhold response to *neutral* expression (20% of stimuli; ‘No-Go'), regardless of the gender of the face (male/female). Stimulus set was balanced for gender, such that there were equal numbers of male and female pictures for each emotion category.

Implicit emotion (IE): participants were asked to respond to pictures of male faces (80% of stimuli; ‘Go') and inhibit response to female faces (20% of stimuli; ‘No-Go'), regardless of their emotional expression. The stimulus set included pictures of happy, neutral and sad expression, with equal probabilities.

### Data analysis

The collected data was processed using IBM SPSS statistic software, version 24. Descriptive statistics were used to analyze the population characteristics, the questionnaires and the cognitive tasks data. Cronbach's Alpha was calculated to ensure the internal reliability of the questionnaires for our study population. We calculated error rate as percentage of errors in No-Go trials (number of No-Go errors/total number of No-Go trials * 100) and average reaction time (RT) to correct Go trails for each task variation and derived it separately for each emotion (happy, sad, and neutral) for the two task variations that included emotions (implicit and explicit emotional variations). We used Signal Detection Theory (SDT) analysis^92^ to calculate task difficulty for each task variation: we derived discrimination accuracy, d’ (Z(hit) − Z(FA)), and criterion (c; − ((Z(hit) + Z(FA))/2); Repeated measures ANOVA was used to compare performance on the three GNG task variations.

Then, Pearson correlations were used to calculate the correlation between PHQ-9 scores and task performance (error rate percentage in No-Go trials and mean reaction time in Go trials) on each of the task variations. In addition, in order to test the strength of association between each task variation and PHQ-9, we used linear regression in which PHQ-9 score was predicted from age and the 3 IC task performance indices (error rate percentage in No-Go trials of NE, IE and EE tasks).

#### Indirect effects analysis

We then tested whether there was an indirect effect of RRS between inhibitory control and depression (PHQ-9). In these models, task performance served as the independent variable, RRS as the proposed indirect effect, and PHQ-9 as the dependent variable. Statistical significance was evaluated through bias-corrected bootstrapping of the product of coefficients, with 5,000 replications. RRS was considered to have an indirect relationship between task performance and PHQ-9 if the bias-corrected bootstrapped confidence intervals of the indirect effect did not contain zero. This approach is considered to have superior power for cross-sectional mediation models^57^.

## Supplementary Information

Supplementary Information.
