# Supplementary material for: The association between implicit and explicit affective inhibitory control, rumination and depressive symptoms
Source: Sci Rep. 2021 Jun 1;11:11490. doi: 10.1038/s41598-021-90875-3 (PMC8169859; doi:10.1038/s41598-021-90875-3)
Supplement: Supplementary file 1 — Supplementary Information. [file 41598_2021_90875_MOESM1_ESM.pdf]

Supplementary Material

Title: The Association between Implicit and Explicit Affective Inhibitory Control, Rumination and Depressive Symptoms

Orly Shimony[1], Noam Einav[1], Omer Bonne[,2], Joshua T. Jordan[3], Tom Van Vleet[4], Mor Nahum[1,*]

[1] School of Occupational Therapy, Faculty of Medicine, Hebrew University, Jerusalem, Israel

[2] Department of Psychiatry, Hebrew University-Hadassah Medical Center, Jerusalem, Israel

[3] Dominican University of California, San Rafael, CA

[4] Department of Research & Development, Posit Science Corporation, San Francisco, CA, USA

Supplemental Material 1. **Application development for web-based data collection**

Online web application development.

A web application for surveys and psychology tasks was developed using javascript, HTML, CSS and JSpsych library. Apache web server was installed on the client-side, serves two Amazon Elastic Compute Cloud (Amazon EC2) instances running our web application. PHP and MySql set up in the backend side. Collected data was transferred to a secure Amazon S3 bucket (datastore service).

In order to collect reaction time (RT) data using web application, we used Amazon CloudFront, a fast content delivery service (CDN) that securely delivers data, videos, application and APIs to customers globally with low latency and high transfer speeds. By using AWS CloudFront, the frontend content is cached in close proximity to the end users, resulting in minimal latency and much better user experience. AWS Elastic Load Balancing was used to automatically distributes incoming application traffic across multiple targets. This allowed us to handle the varying load of application, while distributing the traffic across multiple Availability Zones to ensure the platform's availability.

Data Assurance Protocol.

Since all data in the study was collected remotely, we have taken multiple steps to assure the validity of the data, per recommendations given in the literature (see Berinsky et al., 2012; Walters et al., 2018). Below we detail the main steps taken:

1. First, we used reCAPTCHA (von Ahn L ey al., Science, 2008) to prevent robots from accessing our research.

2. The platforms used to collect online data (MTurk and Prolific) limit the number of daily studies a participant can take part in as a means of combating the conduct that violates the participation agreement (e.g., using robots, scripts or other automated methods to complete the tasks; see https://www.mturk.com/worker/participation-agreement) and to ensure that participants are exercising their independent, human judgement to complete tasks.
3. To ensure cleanliness of the data, and following suggestions in recent publications related to studies using online platforms (e.g., Berinsky et al., 2012; Paolacci & Chandler, 2014; Peer et al., 2014; Walters et al., 2018), the following selection criteria were employed: (a) Residents of the United States (for MTurk participants) or United Kingdom (for Prolific Academic participants) ; (b) Have the "Master's Certification" - workers that consistently demonstrated a high degree of success in performing a wide range of Human Intelligence Tasks (HIT) across a large number of requesters; (c) Approval Rate of >= 95% of their HITs and (d) Over 1000 HITs approved.
4. In order to ensure that the participants are humans and not computer bots and participant marked answers regardless of the questions, we included 5 verification questions throughout the experiment (e.g., “What is the result of 20 X 4?”; “What is your workerID?”; “What stage are you at the experiment?”), as recommended in other studies (see Ahler, Roush & Sood, 2019; Ophir et al., 2019).
5. Finally, we collected response time (RT) data for each questionnaire and task, which provided another layer of confidence on whether participants preformed the tasks as intended.

Supplementary Table 1

Supplemental Table 1. Mental health information about study participants.

Question	Y	N
Have you tried to access mental health support on the NHS in the last 12 months?	57.9%	42.1%
Do you have – or have you had – a diagnosed, on-going mental health/illness/condition?	35.96%	64.04%
Are you currently taking any medication to treat symptoms of depression, anxiety or low mood (e.g. SSRIs)?	36.84% (33.33% anti-depression; 0.88% anti-anxiety; 2.63% combination)	63.16%

Supplementary Table 2. Indirect analyses of the 3 RRS factors (Reflection, Brooding and Depression).

IV	Indirect Variable	$\alpha\beta$ (95% BCI)
Non-Emotional	Reflection	0.039 (0.003, 0.094)*
Implicit Emotion	Reflection	0.050 (0.014, 0.100)*
Explicit Emotion	Reflection	0.047 (0.014, 0.091)*
Non-Emotional	Brooding	0.087 (0.042, 0.150)*
Implicit Emotion	Brooding	0.082 (0.033, 0.144)*
Explicit Emotion	Brooding	0.070 (0.028, 0.124)*
Non-Emotional	Depression	0.127 (0.062, 0.207)*
Implicit Emotion	Depression	0.137 (0.067, 0.214)*
Explicit Emotion	Depression	0.116 (0.060, 0.176)*

Note. IV = Independent Variable, $\alpha\beta$ = product of coefficients to test the indirect effect; BCI = Bias-Corrected Bootstrap Confidence Intervals with 5,000 replications. *$p < 0.05$.
